# Patient and physician characteristics affect adherence to screening mammography: A population-based cohort study

**DOI:** 10.1371/journal.pone.0194409

**Published:** 2018-03-27

**Authors:** Daniela Katz, Angela J. Tengekyon, Natan R. Kahan, Ronit Calderon-Margalit

**Affiliations:** 1 Institute of Oncology, Assaf Harofeh Medical Center, Zrifin, Israel; 2 The Joseph H. and Belle R. Braun Hebrew University-Hadassah School of Public Health and Community Medicine, Jerusalem, Israel; 3 Medical Division, Leumit Health Services, Tel-Aviv, Israel, School of Public Health, Sackler Faculty of Medicine, Tel-Aviv University, Tel-Aviv, Israel; University of Toronto, CANADA

## Abstract

**Background:**

Screening mammograms are widely recommended biennially for women between the ages of 50 and 74. Despite the benefits of screening mammograms, full adherence to recommendations falls below 75% in most developed countries. Many studies have identified individual (obesity, smoking, socio-economic status, and co-morbid conditions) and primary-care physician parameters (physician age, gender, clinic size and cost) that influence adherence, but little data exists from large population studies regarding the interaction of these individual factors.

**Methods:**

We performed a historical cohort study of 44,318 Israeli women age 56–74 using data captured from electronic medical records of a large Israeli health maintenance organization. Univariate analysis was used to examine the association between each factor and adherence (none, partial or full) with screening recommendations between 2008–2014. Multivariate analysis was used to examine the significance of these factors in combination, using binary and multinomial logistic regression.

**Results:**

Among 44,318 women, 42%, 43% and 15% were fully, partially and non-adherent to screening recommendations, respectively. Factors associated with inferior adherence identified in our population included: smoking, obesity, low body weight, low socio-economic status, depression, diabetes mellitus and infrequent physician visits, while, women with ischemic heart disease, female physicians, physicians between the ages of 40 and 60, and medium-sized clinics were associated with higher screening rates. Most factors remained significant in the multivariate analysis.

**Conclusions:**

Both individual and primary-care physician factors contribute to adherence to mammography screening guidelines. Strategies to improve adherence and address disparities in mammography utilization will need to address these factors.

## Introduction

Breast cancer is the leading malignancy in women worldwide [1–3]. Israel has one of the highest incidence rates of breast cancer in the world [4] with about 4,500 women diagnosed annually and 900 women succumbing to the disease [5].

Early diagnosis has been recognized as the most effective tool to improve breast cancer survival [6]. Currently, most guidelines recommend biennial mammography screening (MS) for women aged 50–74 years [5, 7–8].

Multiple factors have been associated with adherence to MS guidelines, including age [1], marital and socio-economic status (SES) [10–11], primary physician's gender and age and practice size that impacts participation in screening programs [12–17].

Under the Israeli National Health Insurance law, all Israeli citizens are insured by one of four Health Maintenance Organizations (HMOs). The "basket" of services provided by these HMOs include free biennial breast cancer screening with mammography to women aged 50–74 years at average-risk to develop breast cancer [18]. Adherence to screening mammography guidelines in Israel is among the highest in westernized countries. The Organization for Economic Co-operation and Development (OECD) reported in its 2011 annual report that, MS rates in Israel were higher than the OECD's average (72.9% vs. 61.5%) [5].

Despite the high MS rate in Israel, about a quarter of eligible women do not comply with these guidelines. The purpose of this study was to identify individual and primary-care physician related factors associated with suboptimal breast cancer screening in a large managed-care population.

## Methods

### Study setting design and population

The study protocol was approved by Assaf Harofeh Medical Center IRB Approval number 0112-15-ASF.

We conducted a population-based cohort study in the setting of Leumit Health Services (LHS), one of the four Israeli HMOs providing medical coverage to ~700,000 members (~8% of the population) nationally. Our study population included 44,730 women aged 56–74 who were insured by LHS continuously between the years 2008–2014. The six year interval allowed at least 3 screening mammography examinations. Women who were diagnosed with breast cancer (n = 412) before or during the six-year study period were excluded from this analysis, leaving 44,318 women in the study.

Data was captured from the electronic Medical registry (EMR) database of LHS using IBM Cognos 10.1.1 BI Report Studio software. Results of queries were downloaded into Microsoft Excel (Version 14) spreadsheets. Data captured included dates of mammography screens done in the past six years, individual factors and primary practitioner factors.

### Study variables

Adherence was classified into three categories: 1. non-adherence, consisting of women with no mammography performed during the study period; 2. partial adherence, consisting of those women who had received one or two mammograms but not the three recommended by the screening guidelines; 3. full adherence, consisting of those women who had received three or more mammograms during the study period. Independent variables were: patient age in years [categorized into four groups (56–59, 60–64, 65–69 and 70–74) for some analyses and treated as a continuous variable for others], marital status (married, divorced, widow and single), body mass index (BMI) [underweight (<18.5), normal (18.5–24.99), overweight (25–29.99) and obese (≥30)], SES status [data from the Central Bureau of Statistics, based on address of residence, ranked on a scale of 1–20 based on housing density, employment, income, education, etc., then collapsed into three categories (low: 1–8, middle: 9–14, and high: 15–20)], smoking status (current smoker vs. non-smoker), diabetes mellitus (DM) (present/absent), depression (present/absent), ischemic heart disease (IHD) (present/absent), number of clinic visits during the six-year period (number of times the patient was seen in the primary-care facility, categorized into < 30, 30–60 and >60), physician age (categorized into four groups: <40, 40–60, >60), physician gender (male/female) and clinic size [(according to the number of registered clients: small = < 1000 clients, medium = 1000 to 4999, large = > 5000, and Other = solo practice physician (mostly serving the Israeli-Arab population)].

#### Data analysis

Descriptive statistics were used to describe the distribution of adherence, socio-demographic factors and co-morbidities among the women by absolute numbers and percentages. Some variables were missing from 30% or more of records, including BMI, smoking status and marital status, so an additional category of “unknown” was introduced to facilitate analysis of these categories in multivariate analysis. The chi-square test was used to evaluate associations between each independent variable and adherence, and multivariate analysis with binary and multinomial logistic regression models was performed to investigate the adjusted association between adherence (any mammography vs. none and partial and full adherence vs. none, respectively) and the study covariates. For logistic regressions we report odds ratios (OR), 95% confidence intervals (CI) and two sided p-values. Statistical package for the social sciences (SPSS) version 20.0 for Windows was used for all analyses.

## Results

A total of 44,318 women with a median age of 63 years met the study inclusion criteria. Of these women, 42% were fully adherent, 43% were partially adherent, and 15% received no mammograms during the study period. The distribution of the individual characteristics of the study population and of the treating physician by adherence to screening mammography is presented in Tables 1 and 2, respectively.

**Table 1:** Distribution of individual characteristics of the study population by adherence to mammography.

Variables	Non-adherent N = 6770	Partially Adherent N = 19092	Fully Adherent N = 18456	P-value
**Patient Age**				** **
56–59	1976 (29.2%)	5806 (30.4%)	4957 (26.9)	** **
60–64	2128 (31.4%)	6230 (32.6%)	6009 (26.9%)	
65–69	1710 (25.3%)	4643 (24.3%)	5287 (28.6%)	<0.001
70–74	956 (14.1%)	2413 (12.6%)	2203 (11.9%)	
**Marital Status**				
Married	2565 (37.9%)	8023 (42%)	8653 (46.9%)	
Divorced	795 (11.7%)	2317 (12.1%)	2186 (11.8%)	<0.001
Widow	355 (5.2%)	938 (4.9%)	876 (4.7%)	
Single	542 (8%)	890 (4.7%)	779 (4.2%)	
Missing	2513 (37.1%)	6924 (36.3%)	5962 (32.3%)	
**SES**				
Low	2515 (37.1%)	6334 (33.2%)	4552 (24.7%)	
Middle	3350 (49.5%)	10124 (53.0%)	11129 (60.3%)	
High	482 (7.2%)	1492 (7.8%)	1846 (10.0%)	<0.001
Missing	423 (6.2%)	1142 (6.0%)	929 (5.0%)	
**Smoking**				
Non smokers	2999 (44.3%)	10530 (55.2%)	11591 (62.8%)	
Current smokers	597 (8.8%)	1812 (9.5%)	1345 (7.3%)	<0.001
Missing	3174 (46.9%)	6777 (35.5%)	5520 (29.9%)	
**BMI**				
Underweight	36 (0.5%)	98 (0.5%)	51 (0.3%)	
Normal	711 (10.5%)	2307 (12.1%)	2292 (12.4%)	
Overweight	838 (12.4%)	3079 (16.1%)	3115 (16.9%)	<0.001
Obese	964 (14.2%)	3212 (16.8%)	2650 (14.4%)	
Missing	4221 (62.3%)	10396 (54.5%)	10308 (55.9%)	
**Clinic Visits**				
<30	2386 (37.1%)	3113 (16.3%)	1301 (7%)	
31–60	1831 (28.4%)	5939 (31.1%)	5136 (27.8%)	<0.001
>60	2222 (34.5%)	10022 (52.5%)	12018 (65.1%)	
**DM**	1294 (19.1%)	4548 (23.8%)	4007 (21.7%)	<0.001
**IHD**	646 (9.5%)	2893 (15.2%)	2962 (16%)	<0.001
**Depression**	608 (9%)	1844 (9.7%)	1902 (10.3%)	<0.005

The number and percentage for each independent variable is shown for the dependent variable groups non-adherent, partially adherent and fully adherent. The p values represent the significance of the specific factor in univariate analysis.

**Table 2:** Distribution of physician and clinic characteristics by adherence to mammography.

Variables	Non-adherent	Partially Adherent	Fully Adherent	TOTAL	P-value
**MD age**					
< 40	251 (3.7%)	634 (3.3%)	355 (1.9%)	1240 (2.8%)	
40–60	4209 (62.2%)	12649 (66.3%)	13090 (70.9%)	29948 (67.6%)	<0.001
60–80	1925 (28.4%)	5470 (28.7%)	4861 (26.3%)	12256 (27.7%)	
Missing	385 (5.7%)	339 (1.8%)	150 (0.8%)	874 (2%)	
TOTAL	6770	19092	18456	44318	
**MD gender**					
Male	3313 (48.9%)	9317 (48.8%)	7977 (43.2%)	20607 (46.5%)	
Female	3097 (45.7%)	9490 (49.7%)	10370 (56.2%)	22957 (51.8%)	<0.001
Missing	360 (5.3%)	285 (1.5%)	109 (0.6%)	754 (1.7%)	
TOTAL	6770	19092	18456	44318	
**Clinic size**					
Small	232 (3.4%)	686 (3.6%)	558 (3%)	1476 (3.3%)	
Medium	2530 (37.4%)	8580 (44.9%)	8501 (56.2%)	19611 (51.8%)	<0.001
Large	3480 (51.4%)	8611 (45.1%)	8721 (47.3%)	20812 (47%)	
Other	498 (7.4%)	1126 (5.9%)	573 (3.1%)	2197 (5%)	
Missing	30 (0.4%)	89 (0.5%)	103 (0.6%)	222 (0.5%)	
TOTAL	6770	19092	18456	44318	

The number and percentage for each independent variable is shown for the dependent variable groups non-adherent, partially adherent and fully adherent. The p values represent the significance of the specific factor in univariate analysis.

### Adherence to mammography screening

Low SES was associated with low performance of mammography. Divorced, widowed and single women were less likely than married women to have any mammography (OR of 0.88, 0.83, and 0.49, respectively). Non-smoking was associated with increased odds of having any mammography (OR: 1.41; 95% CI: 1.28–1.55). Compared with healthy-weight women, those who were underweight had decreased odds of any mammography (OR:0.56; 95% CI: 0.37–0.85), whereas the other weight categories had no significant association. Controlling for the other comorbidities and number of clinic visits, DM and depression were associated with non-performance of mammography (DM, OR: 0.74, 95% CI:0.69–0.80; Depression, OR: 0.79, 95% CI:0.72–0.87) whereas IHD was positively associated with performance of mammography (OR:1.18, 95% CI:1.08–1.29). The number of clinic visits were associated with adherence to mammography (<30, 30–60 visits per study period OR: 0.17 and 0.58, respectively, compared with >60 visits per year).

### Level of adherence to mammography screening

In a multivariate analysis of the association of all individual factors with level of adherence (Table 3), the odds of low SES women to undergo 1–2 MS were low (OR: 0.71, 95% CI:0.63–0.8) and their odds to be adherent to biennial mammography were further diminished (OR:0.40, 95% CI:0.35–0.45). Similarly, non-smoking was associated with superior mammography performance levels with a stronger association for full adherence (OR:1.64, 95% 1.46–1.84; p<0.001) than for partial adherence (OR:1.10, 95% (0.99–1.23; p = 0.06). In general, BMI was not associated with partial adherence, however, both being underweight and obese were negatively associated with full adherence (OR: 0.49, 95% CI: 0.30–0.97, p = 0.004 and OR: 0.77, 95% CI: 0.68–0.88, p<0.001, respectively).

**Table 3:** Association between individual characteristic variables and adherence to mammography screening recommendations over the six-year period.

		Partial adherence vs. non-adherence			Full adherence vs. non-adherence		
**Variable**		**OR**	**95%CI**	**P**	**OR**	**95%CI**	**P-value**
**Age**		0.965	0.959–0971	<0.001	0.967	0.961–0.973	<0.001
**BMI**	Underweight	0.862	0.567–1.310	0.486	0.493	0.305–0.794	0.004
	Normal	**1**			**1**		
	Overweight	1.112	0.986–1.255	0.85	1.156	1.021–1.309	0.022
	Obese	0.934	0.830–1.052	0.261	0.773	0.684–0.875	<0.001
	Unknown	0.83	0.751–0.918	<0.001	0.875	0.790–0.970	0.011
**SES**	Low	0.71	0.631–0.800	<0.001	0.398	0.353–0.449	<0.001
	Medium	0.939	0.837–1.053	0.283	0.836	0.745–0.938	0.002
	High	**1**			**1**		
**Smoking**	Non-smokers	1.109	0.995–1.236	0.061	1.638	1.461–1.837	<0.001
	Current smokers	**1**			**1**		
	Unknown	0.908	0.813–1.014	0.086	1.034	0.919–1.163	0.576
**DM**	Yes	0.94	0.869–1.016	0.121	0.72	cl	<0.001
	No	**1**			** 1**		
**Depression**	Yes	0.801	0.723–0.887	<0.001	0.767	0.691–0.851	<0.001
	No	**1**			**1**		
**IHD**	Yes	1.325	1.201–1.461	<0.001	1.269	1.150–1.401	<0.001
	No	**1**			**1**		
**Clinic visits**	<30	0.266	0.245–0.288	<0.001	0.085	0.078–0.094	<0.001
	30–60	0.681	0.632–0.734	<0.001	0.458	0.425–0.494	<0.001
	>60	**1**			**1**		

The results of a multinomial logistic regression analysis are shown, with non-adherence as the reference category. Since, adherence decreased with age; the remaining variablesare age-adjusted. The reference category for the dependent variables was set as non-adherence. The reference category within each independent variable was set to an odds ratio of one.

The number of clinic visits was strongly associated with level of adherence; Women who had less than 30 visits over 6 years of follow up had an OR of 0.27 for partial adherence and OR of 0.08 for full adherence, compared to women who had more than 60 visits during the same time. Controlling for number of clinic visits, each of the comorbidities evaluated was associated differently with adherence to mammography. The associations of IHD and depression were not modified by level of adherence. Women diagnosed with depression had similarly reduced odds to have either partial or full adherence to mammographic screening (OR: 0.80, 95% CI: 0.72–0.88; p<0.001, and OR: 0.76, 95% CI: 0.7–0.85; p<0.001, respectively), whereas women diagnosed with IHD had similarly increased odds for both partial and full adherence (OR: 1.32, 95% CI: 1.2–1.46; p<0.001, and OR: 1.27, 95% CI: 1.15–1.4; p<0.001, respectively). Women with DM had decreased probability to be fully adherent (OR: 0.72, 95% CI: 0.66–0.78; p<0.001), however, DM was not associated with partial adherence.

In the multivariate analysis of the characteristics of the primary health provider (Table 4) gender and age of the primary physician were strongly associated with women adherence. Full adherence was negatively associated with male physicians (OR:0.70, 95% CI: 65–0.73; p<0.001) and with physicians younger than 40 years (OR: 0.53, 95% CI: 0.44–0.63; p<0.001). Solo practicing physicians were less likely to have patients with full adherence (OR: 0.46, 95% CI: 0.42–0.49; p<0.001). In summary, the highest adherence was noted among female physicians, physicians that that were 40–59 years old, and medium size clinics.

**Table 4:** Association between characteristics of the primary health provider and adherence over a six-year period.

Variable		Partial Adherence			Full Adherence		
		OR	95%CI	p-value	OR	95%CI	p-value
Physician Age	<40	0.821	0.699–0.965	0.017	0.530	0.443–0.635	<0.001
	40–60	1.067	1.000–1.139	0.050	1.246	1.165–1.333	<0.001
	>60	**1**	–	–	**1**	–	–
Physician sex	Male	0.866	0.816–0.920	<0.001	0.691	0.650–0.735	<0.001
	Female	**1**	–	–	**1**	–	–
Size of clinic	Small	1.091	0.928–1.283	0.290	0.880	0.742–1.042	0.139
	Medium	1.278	1.201–1.361	<0.001	1.205	1.130–1.285	<0.001
	Large	**1**	–	–	**1**	–	–
	Solo practicing	1.142	0.992–1.316	0.065	0.592	0.507–0.691	<0.001

Model was adjusted in addition to patients' age and number of clinic visits

The results of a multinomial logistic regression analysis are shown, with non-adherence as the reference category. Age was associated with worse adherence as patients became older; the remaining results in the table are age-adjusted. The reference category for the dependent variables was set as non-adherence. The reference category within each independent variable was set to an odds ratio of one.

In a model where characteristics of both individuals and primary health providers were included, results remained similar (S1 Table).

## Discussion

To our knowledge this is the first large scale, population-based, long term study that investigated both individual and primary-care physician parameters associated with adherence to current guidelines for mammography. Altogether, 42% complied with MS guidelines whereas 43% were only partially adherent and 15% received no mammograms during the study period. The combined rates of "full" and "partial" adherence in our study compare favorably with other administrative data based studies. Ulcickas et al. [19] found that among 8749 women 50–74 years old, 88% performed a mammography within 5 years of the initial screening. However, higher screening rates in self-reported studies such as the study reported by Rakowski et al. [20] (81% screening within 2 years of the initial screening and 72% repeated screening within 4 year of the initial mammography) were probably biased by recall or simply by imperfect time-recollection underestimating the time elapsed since the previous screening [21].

Our study incorporated a novel extended-follow up design that included evaluation of both "partial" and "full" adherence to screening over six years of follow up as opposed to previous studies that were limited to dichotomous analysis of adherence vs non-adherence over two years [19, 22–26], on-schedule vs not on-schedule [27–28] or much smaller studies that collected data on recent vs repeat mammography over 4 years of follow up relying on self-reported answers [20].

We observed that while older age, high BMI, low SES, smoking, DM, depression, and male gender of the primary-care physician were associated with decreased full adherence, increased full adherence was found to be associated with increased number of clinic visits, IHD, mid-aged physician (40–59 years old) and medium-sized clinics. Some factors were associated with both partial and full adherence (older age, low SES, depression, fewer clinic visits, male physician gender, mid-aged physician (40–59 years old) and medium-sized clinics). This is in line with several other studies advocating past behavior as generally a strong predictor of future behavior [20, 23], and therefore relatively similar characteristics are associated with both performance-frequencies.

Whereas, for early detection partial adherence to mammography guidelines is still better than no MS, partial adherence is less contributory to early cancer detection and poses an increased risk for missing growing tumors [23]. Factors associated with the health-provider such as confusion about screening guidelines and low perceived self-cancer risk were among the characteristics described by Halabi et al to be associated with delayed screening [27]. Both attributes may project the primary-care provider attitude towards screening. Thus it is not surprising that several studies reported that of all factors, the absence of a provider recommendation was the most important attribute affecting repeat screening mammography in insured women with universal coverage [13, 27,29].The primary-care physician may settle the above in-clarity not only by motivating a woman to undergo screening but also reinforcing repeated on-schedule screening [23].

Screening mammography recommendations were found to be associated with gender and age among primary-care physicians. Female physicians endorse MS guidelines more frequently than male physicians [12]. Like other studies, we found female physicians had better adherence to screening recommendations [12–14, 30–31]. Interestingly, one report shows that these gender gaps occur early in physicians’ carrier. Among medical fellows, repeat mammography rates were higher for women treated by female fellows [30].

We found that non-solo practices were positively associated with MS. The impact of the clinic setting on adherence to mammography is supported by the finding that practices with a female-gender orientation, such as women's health groups and Obstetrics and Gynecology clinics, had much higher referrals to mammography and repeat mammography, compared with general internal medicine services had higher referral rates [30, 32]. Our results suggest that interventions aimed at young, male physicians and those who work in solo practices may yield the greatest effect in increasing adherence. Such interventions could include seminars, mail alerts or incentives to drive physicians to refer patients to mammography.

Availability of health insurance is probably the most important contributor to adherence to screening in the US and countries without universal health insurance [9, 20, 28, 33, 34]. The universal healthcare in Israel covers screening mammography. Our results of low utilization of MS among women with low SES despite the universal coverage are comparable with statistics of the national program for community quality indicators as well as results of a previous study that included 158,000 women insured by another HMO in Israel (OR = 0.79, 95% CI 0.76–0.81) [22]. Similarly, these results are supported by reports from Canada, France, UK, and Sweden, where cost of MS is not a barrier either to women or physicians [35–37].

Smoking is commonly associated with low SES, unhealthy lifestyle, stress and mood disorders [24]. While previous studies indicate that smokers undergo less screening mammography [38–39], our results demonstrate that smoking is negatively associated only with adherence to the biennial schedule (full adherence), meaning that in our population smoking was not an obstacle to screening and only a hurdle for on-schedule mammography.

With respect to obesity and overweight, studies indicate that obesity has been positively associated with breast cancer risk and breast cancer recurrence and therefore MS is particularly timely in this subgroup [40–42]. Similar to one other study [25], we found that obese and underweight women, on both BMI extremes, defer and do not utilize mammography according to guidelines recommendations. Mammography can be more cumbersome in obese women as more films are required to cover the entire breast [43], and cause discomfort to underweight women without much breast tissue to compress [25]. In addition, both BMI extremes may be caused by an underlying illness responsible for poorer general health compared with normal BMI women, which may result in delaying screenings [25, 42, 44].

Results are mixed for mammography use among women with comorbidities. One study found that DM by itself was not a detrimental factor for screening, although DM related parameters such as DM quality of care, better health status and higher SES better correlated with higher odds of mammography performance [45]. Other studies indicate an inverse association between DM, IHD and depression and MS [45–47]. Clinic visits served as a surrogate for the interaction-time with the health provider. After controlling for number of visits, women with IHD were more likely to comply with mammography (partial adherence OR:1.33, 95% CI: 1.20–1.46; Full adherence OR: 1.27, 95% CI: 1.15–1.40), and women with depression were less likely to comply (OR: 0.80, 95% CI: 0.72–0.89, and OR:0.77, 95% CI:0.69–0.85, respectively).

We did not examine the effect of two or more comorbidities on mammography performance as Orenstein et al [48], who report, a curvilinear relationship with number of simultaneous chronic conditions, and an increased likelihood of being up to date with mammography as the number of chronic conditions increased from 0 to 4 or 5. It is therefore seems advisable to create outreach programs aimed at specific populations such as those with underweight, smokers and women with diabetes mellitus or depression. The negative association with number of clinic visits emphasizes the need for outreach programs, that may include interventions at smoking cessation programs or dedicated diabetes clinics.

The strength of this study relies on the fact that we had access to six years of data enabling the capture of three screening events, whereas most previous studies generally relied on two years of data. Furthermore, since we relied on claims data rather than self-reporting or referrals, we only captured mammograms that were in fact performed and avoided both the recall and non-performance biases (patients not screened despite documentation of referral). Additionally, since this study was conducted in a national managed-care system, we avoided loss to follow up from relocation within the country.

This study is therefore unique as the data source came from a national healthcare service. It permitted a parallel comprehensive evaluation of individual characteristics along with healthcare attributes in a large population of insured women.

This study had several limitations. Although we were able to capture diseases based on the diagnosis in the EHR, we were unable to include severity of illness and physicians may not have registered all diagnosed diseases. The data on individual factors was not always available.

## Conclusions

Our study helps highlight the complex interaction between individual and primary-care physician factors that influence adherence. With a better understanding of these factors, physicians and those responsible for public and corporate policy can examine the processes for which they are responsible, focusing efforts where they can have the greatest impact for improving MS adherence, reducing the morbidity and mortality from breast cancer.

## Supporting information

S1 Table (DOCX)
