# Supplementary material for: Patient and physician characteristics affect adherence to screening mammography: A population-based cohort study
Source: PLoS One. 2018 Mar 27;13(3):e0194409. doi: 10.1371/journal.pone.0194409 (PMC5870964; doi:10.1371/journal.pone.0194409)
Supplement: S1 Table — (DOCX) [file pone.0194409.s001.docx]

|  |  | | Partial Adherence | | | | Full Adherence | | |
| --- | --- | --- | --- | --- | --- | --- | --- | --- | --- |
|  | |  | OR | 95% CI | p-value | OR | | 95% CI | p-value |
| Age | |  | .968 | .961- .974 | <0.001 | .968 | | .962-.975 | <0.001 |
| BMI | | Underweight | .931 | .604-1.430 | .747 | .542 | | .332-.886 | 0.015 |
|  | | Normal | 1 |  |  | 1 | |  |  |
|  | | Overweight | 1.105 | .978-1.250 | .108 | 1.152 | | 1.020-1.310 | 0.027 |
|  | | Obese | .916 | .812-1.03 | .150 | .767 | | .677-.869 | <0.001 |
|  | | Unknown | .841 | .760-.930 | .001 | .887 | | .799-.985 | 0.024 |
| SES | | Low | .772 | .684-.872 | <0.001 | .459 | | .405-.520 | <0.001 |
|  | | Medium | .977 | .869-1.100 | .703 | .852 | | .757-.959 | <0.001 |
|  | | High | 1 |  |  |  | |  |  |
| Smoking | | Non-smokers | 1.120 | 1.040-1.190 | <0.001 | 1.650 | | 1.54-1.770 | <0.001 |
|  | | Current smokers | 1 |  |  | 1 | |  |  |
|  | | Unknown | .932 | .894-1.120 | .219 | 1.070. | | .888-1.130 | 0.247 |
| DM | | Yes | .968 | .893-1.05 | .427 | .748 | | .689-.812 | <0.001 |
|  | | No | 1 |  |  | . | | . | . |
| Depression | | Yes | .790 | .712-.877 | <0.001 | .746 | | .672-0.829 | <0.001 |
|  | | No |  |  |  |  | |  |  |
| IHD | | Yes | 1.360 | 1.230-1.500 | <0.001 | 1.31 | | 1.190-1.450 | <0.001 |
|  | | No |  |  |  |  | |  |  |
| Clinic visits | | <30 | .270 | .249-0.294 | <0.001 | .085 | | .077-.093 | <0.001 |
|  | | 30-60 | .680 | .629-.734 | <0.001 | .446 | | .413-.483 | <0.001 |
|  | | >60 | 1 |  |  | 1 | |  |  |
| Physician Age | | <40 | .858 | .727-1.010 | 0.070 | .625 | | .519-0.752 | <0.001 |
|  | | 40-60 | 1.084 | 1.010-1.160 | 0.018 | 1.286 | | 1.199-1.380 | <0.001 |
|  | | >60 | 1 |  |  |  | |  |  |
| Physician sex | | Male | .873 | 0.820-0.928 | <0.001 | .725 | | .680-.773 | <0.001 |
|  | | Female | 1 |  |  | . | | . | . |
| Size of clinic | | Small | 1.398 | 1.100-1.770 | 0.005 | 1.027 | | .800-1.320 | 0.834 |
|  | | Medium | 1.262 | 1.180-1.350 | <0.001 | 1.203 | | 1.130-1.290 | <0.001 |
|  | | Large | 1 |  |  |  | |  |  |
|  | | Solo practicing | 1.243 | 1.070-1.440 | 0.003 | .757 | | .644-.888 | 0.001 |

S1 Table: **Joint multivariate model** of individual and primary health provider characteristics for adherence to mammography. OR, odds ratio; CI, confidence interval; BMI, body mass index; SES, socioeconomic status; DM, diabetes mellitus; IHD, ischemic heart disease;
